# The functional variant of *NTN1* contributes to the risk of nonsyndromic cleft lip with or without cleft palate

**DOI:** 10.1038/s41431-019-0549-4

**Published:** 2019-11-28

**Authors:** Dandan Li, Guirong Zhu, Shu Lou, Lan Ma, Chi Zhang, Yongchu Pan, Lin Wang

**Affiliations:** Jiangsu Key Laboratory of Oral Diseases, Nanjing Medical University, Nanjing, 210029 China

**Keywords:** Genetic predisposition to disease, Risk factors, Gene expression

## Abstract

Previous genome-wide association study of nonsyndromic cleft lip with or without cleft palate (NSCL/P) identified a susceptible variant (rs4791774). We hypothesized that the functional single nucleotide polymorphism (SNP) may be in linkage disequilibrium with this lead SNP. The potential functional SNP (rs4791331) was identified by bioinformatic analysis. A case–control study with 891 orofacial cleft cases and 830 controls was designed to investigate its association with orofacial cleft. The allele-specific DNA-protein binding preference was predicted by JASPAR database. Cell proliferation, cycle and apoptosis, luciferase activity and *netrin-1* (*NTN1*) expression were examined after transfection with the rs4791331 C/T vector in HEK-293 and HEPM cell lines. Forty-six lip tissues of NSCL/P patients were collected to detect *NTN1* expression. *ntn1a* knockout zebrafish models were generated by CRISPR/Cas9 and observed with micro-CT. In the case–control study, the rs4791331-T allele was associated with an increased risk of nonsyndromic orofacial cleft (OR = 1.41, 95% CI = 1.19–1.68), as well as the subgroups cleft lip only (OR = 1.46, 95% CI = 1.14–1.87) and cleft lip and palate (OR = 1.58, 95% CI = 1.27–1.96). The T allele of rs4791331 exhibited anti-apoptotic effects and promoted cell cycle progression at the G1/S transition. Decreased enhancer activity and reduced *NTN1* expression following transfection of the T allele were observed. Carriers of the CT/TT genotypes showed significantly lower expression of *NTN1* than CC carriers. The *ntn1a*^−/−^ zebrafish showed relatively wider intermaxillary fissures. These results indicate that rs4791331 (C > T) disrupted motif binding and led to abnormal expression of *NTN1*, which may be involved in the development of NSCL/P.

## Introduction

Orofacial cleft is the most common congenital craniofacial malformation affecting humans [1], causing severe health-related problems as well as financial burdens for patients and their families [2]. Approximately 30% of orofacial clefts are syndromic and may be inherited in Mendelian fashion, whereas the remaining cases are nonsyndromic and appear to be induced by multiple genetic and environmental factors [3–6]. For nonsyndromic orofacial cleft (NSOC), the most common types are nonsyndromic cleft lip with or without cleft palate (NSCL/P) and nonsyndromic cleft palate only.

Single nucleotide polymorphisms (SNPs) have attracted tremendous interest in recent years as potential susceptibility factors for complex traits. To date, dozens of genomic loci have been identified for NSOC by genome-wide association studies (GWASs) [7–14]. The data provided statistical support for the association between SNP markers and risk of NSOC.

However, the translation from statistical associations revealed by GWASs to the biological causes of NSOC is still a major challenge. The majority of these loci are located within intronic or intergenic regions. Understanding their function in gene regulation has been recognized as the key to connecting these phenotypes with DNA sequence variations. The most frequently affected elements are transcriptional enhancers and silencers, although the connection between sequence and function is currently poorly understood [15]. In most cases, SNPs within noncoding DNA are presumed to disrupt *cis*-regulatory elements [16].

Previous studies showed that SNPs in strong linkage disequilibrium (LD) with the lead SNP could be functional for NSCL/P. As Liu et al. reported, three most-highly risk-associated SNPs at 1p22 were identified with allele-dependent effect, affecting transcription factors binding ability or chromatin configuration [16]. Leslie et al. identified a common non-coding variant, rs227727, in 100% linkage disequilibrium with the most-strongly associated SNP identified in GWAS that alters the function of an enhancer [17]. Cvjetkovic et al. found a functional variant rs138557689 in intron of *FZD6* by sequencing of the linkage region of 8q21.3-24.12, a candidate locus of NSCLP [18]. And, it was supposed to be an etiologic variant in linkage disequilibrium with rs41268753 for the etiology of cleft palate by Leslie et al. [19].

Our group previously performed the first GWAS for NSCL/P in a Chinese population, and rs4791774 (hg19 NC_000017.10:g.8932119 A > G, NC_000017.10:g.8932119 A > C), located at chromosome 17p13.1, showed a genome-wide association with NSCL/P in this study [13] as well as in the targeted sequencing study of Asian case-parent trios [17]. This SNP is located in the intronic region near the transcription start site of netrin-1 (*NTN1*, Nucleotide RefSeq NM_004822.2 and exon numbering NC_000017.10). However, the functional mechanism underlying this association has not been scrutinized. Here, we first filtered the potentially functional SNP based on bioinformatic analysis and investigated its association with the risk of NSCL/P in a case–control cohort and then performed a series of experiments to explore its functional significance.

## Materials and methods

### Bioinformatic analysis

RegulomeDB and F-SNP for functional prediction were applied. HaploReg V4.1 software was used for LD analysis of rs4791774 to identify SNPs in high LD (*r*^2^ ≥ 0.8) with rs4791774 according to the LD information from the Asian population of 1000 Genomes Project Phase 1. The binding abilities of the transcription factors were predicted by JASPAR. The relative expression data of *Ntn1* in the 10.5–14.5-day mouse embryos (period of lip and palate fusion) were downloaded from Gene Expression Omnibus (GEO).

### Human subjects and DNA extraction

This is an ongoing project for genetic study of NSOC, approved by the Ethics Review Committee (NJMUERC [2008] No. 20). The recruitment of study subjects was described preciously(Li et al. 2016). In brief, patients were recruited from three hospitals in Jiangsu Province between August 2008 and January 2015. Physical examination and medical records were obtained for clinical assessment. Patients with congenital isolated oral clefts without syndromic symptoms or other birth defects met the inclusion criteria of the case group. The controls were self-reported Chinese Han from the same region, and no known congenital anomalies were detected. Informed consent was provided by every volunteer of the study.

Approximately 2 ml of venous blood was collected from each participant and stored in a tube containing ethylenediaminetetraacetic acid. Genomic DNA was extracted by the phenol-chloroform method. With the ABI Prism 7900HT system (Applied Biosystems), all samples were genotyped by a TaqMan allelic discrimination method with a call rate of 98.6%. The sequences of the primers and probes are shown in Supplementary Table S1. Approximately 5% of the samples were randomly repeated, and the results were 100% concordant.

### Cell culture

HEK-293 (ATCC-1573^TM^) and HEPM (human embryo palate mesenchyme, ATCC-1486^TM^) cell lines were purchased from the American Type Culture Collection (ATCC, Manassas, VA, USA) and cultured in Eagle’s minimal essential medium with 10% fetal bovine serum (ATCC) and 1% Penicillin-Streptomycin Solution (Gibco, Foster City, CA, USA).

### Plasmid construction and transient transfection

We cloned ~700 bp regions containing the individual alleles of rs4791331 (hg19 NC_000017.10:g.8932082 C > T). A plasmid containing the *NTN1* fragment of the rs4791331 major C allele was inserted downstream of the luciferase gene in the PGL3-promoter vector (Promega, Madison, WI, USA) at the XbaI site. With this construct as the template, the minor allele was generated by the site-specific mutagenesis method. The nucleotide sequence of the recombinant plasmids was verified by DNA sequencing. (Supplementary Fig. S1). The PGL3-promoter vector with a random sequence inserted was used as the negative control.

Plasmids containing the C or T allele were transfected in cells using Lipofectamine 2000 (Invitrogen, Carlsbad, CA, USA).

### Cell proliferation, cycle and apoptosis analysis

While cell culture continued for 24, 48, and 72 h after plasmid transfection, the Cell Counting Kit 8 (Dojindo, Kumamoto, Japan) was used to assess cell proliferation. The absorbance value at 450 nm was determined using a SpectraMAX 190 (Molecular Devices, USA). Absorbance at each time point was tested in triplicate with six independent repeats.

Forty-eight hours after transfection, cells were harvested and fixed overnight in 70% ice-cold ethanol followed by RNase A treatment and stained with 50 μg/ml of propidium iodide for DNA content analysis by flow cytometry on a FACSCalibur system (BD Medical Technology, USA). The results are expressed as a percentage of cells in each cell cycle phase. Cell apoptosis was detected by Annexin V-FITC/PI double staining 48 h after transfection.

### Dual-luciferase reporter assay

The luciferase activity in the lysates was quantified with a dual-luciferase reporter assay system 48 h after transfection (Promega, Madison, WI). The luminescent reaction of the Renilla luciferase was simultaneously activated after the firefly luciferase reporter was measured as a stabilized luminescent signal. The firefly luciferase to Renilla luciferase ratio was considered the relative reporter activity. Independent triplicate experiments were performed for each plasmid construct.

### Lip tissue collection, RNA extraction, and quantitative real-time PCR

Forty-six redundant lip tissue samples from NSCL/P patients who underwent surgery were collected. Total RNA was isolated using TRIzol reagent (Invitrogen, Carlsbad, CA, USA). The *NTN1* mRNA levels relative to the transcription level of *GAPDH* were quantified by quantitative reverse transcriptase-polymerase chain reaction (RT-qPCR) in an ABI 7900 HT instrument (Applied Biosystems, Foster City, CA, USA). All RT-qPCR reactions were performed using SYBR Green Real-Time PCR (TaKaRa, Shiga, Japan) according to the manufacturer’s instructions. All reactions were performed in triplicate, and the relative gene expression was determined by the 2^−ΔΔCt^ method [20]. The primers are listed in Supplementary Table S1.

### Zebrafish models

Although vertebrates boast an enormous diversity of adult facial morphologies, the fundamental signaling pathways and cellular events that sculpt the nascent craniofacial structure in the embryo have proven to be highly conserved from zebrafish to man [21]. The zebrafish, affording experimental advantages toward investigating the normal function of genes associated with orofacial development, has served as a popular model since the 1990s. many researchers have employed this model to explore the functions of genes implicated in cleft lip and cleft palate [16, 22, 23].

According to the synteny analysis (Supplementary Fig. S2) and phylogenetic tree analysis (Supplementary Fig. S3), the *ntn1a* gene of zebrafish was identified as the orthologue of the human *NTN1* gene with 86% similarity in an amino acid alignment. According to previous studies, *ntn1a* expressed in the orofacial region during the facial development [24–26]. Therefore, we supposed that *ntn1a* may affect the orofacial development of zebrafish without detecting its expression. CRISPR was designed to target a site in exon 1 of the *ntn1a* gene. Mutagenesis and founder identification were carried out with the *ntn1a* primers 5’-TTT CGC AAA AGT CCA GGT AGT G-3’ (reverse) and 5’-TGG GTG TGT GTG ACT CCA TAT TG-3’ (forward) (the recognition sequence and target sequence are listed in Supplementary Table S2). 2 μl of sgRNA stock (500 ng/μl) was mixed with 2 μl of recombinant Cas9 protein (1 μg/μl, PNA Bio, Thousand Oaks, CA) and incubated on ice for at least 10 min to allow formation of the sgRNA/Cas9 complex. 2 nl of the injection mix was injected intracellularly in one-cell stage zebrafish embryos using glass needles and a micromanipulator. DNA was extracted from 10 pooled injected embryos and an uninjected control group at 48 h post fertilization using the HotShot protocol. Mutagenesis was determined by a T7 endonuclease assay. Positive clutches (F0 generation) were raised to adulthood and outcrossed against wild-type fish. Germline transmission was also determined by the T7 endonuclease assay. Positive clutches (F1 generation) were raised to adulthood and genotyped individually. Fish carrying the same variant were pooled as the founders of the heterozygous stable knockout line. The F1 generation was incrossed to obtain the homozygous ntn1a knockout F2 generation. Then, 3D images of the 4-month-old F2 generation were taken with a resolution of 18 μm using SkyScan 1276 Micro-CT (Bruker, Germany).

### Statistical analysis

Statistical analyses were performed using Version 9.1 of the SAS^®^ software (SAS Institute Inc., Cary, NC, USA). For all graphs, data are shown as the mean ± standard deviations (SD). An independent-sample *t* test was applied for the comparison of different groups. The demographic characteristics of cases and controls were analyzed by Chi-square (χ^2^) tests. Hardy–Weinberg equilibrium (HWE) of genotype frequencies in the control group was tested by Fisher’s exact test. Odds ratios (ORs) and 95% confidence intervals (CIs) for risk of NSOC were calculated using logistic regression analyses. All *P* values were two-sided. The RNA-Seq count data with gene assignments downloaded from the GEO database were read and visualized by R (version 3.4.2) (R Core Team, 2017) and analyzed by one-way ANOVA.

All the variant data has been submitted to ClinVar (Submission ID: SUB6440632, Organization ID: 6434903).

## Results

### Identification of the potentially functional SNP by in silico analysis

The score identifying regulatory elements by RegulomeDB and F-SNP was 4 (minimal binding evidence) and 0.194, respectively, for rs4791774 (Supplementary Table S3), indicating this index GWAS biomarker signal had yet unidentified causal variants. Instead of examining the lead SNP, one optional strategy is to analyze the functional SNPs in high LD with the lead SNP that modify the incidence of birth defects [27].

LD analysis showed that rs4791331 (*r*^2^ = 0.99), rs9891446 (*r*^2^ = 0.8), and rs36047638 (*r*^2^ = 0.8) were highly linked with rs4791774. Among them, rs4791331 was the functional site with the highest potential, with values of 2b by RegulomeDB and 0.714 by F-SNP (Supplementary Table S3); the two software programs consistently predicted that rs4791331 was a transcription factor binding site.

### Association of rs4791331 and risk of NSCL/P

A total of 856 unrelated NSOC cases and 823 healthy controls were recruited for this study. As shown in Table 1, the case group consisted of three subgroups: 266 cases of cleft lip only (CLO), 381 cases of cleft lip and palate (CLP), and 226 cases of cleft palate only (CPO). No statistical discrepancy in gender was identified between the cases and controls.

**Table 1:** Demographic characteristic information of the study samples

	Cases (*N* = 873)		Controls (*N* = 830)		*P* ^a^
	*N*	%	*N*	%	
Gender					
Male	534	61.2	471	56.7	0.068
Female	339	38.6	359	43.3	
Cases subtype					
CLO	266	30.5			
CLP	381	43.6			
CPO	226	25.9			

*CLO* cleft lip only, *CLP* cleft lip with cleft palate, *CPO* cleft palate only

^a^Two-sided chi-square test

The distribution of three genotypes in the control group was consistent with HWE (*P* = 0.412). As presented in Table 2, the T allele was associated with an increased risk of NSOC (OR = 1.43, 95% CI = 1.20–1.70, *P* = 5.10 × 10^−5^, additive model). In the subtype analysis, the T allele was associated with CLO (OR = 1.46, 95% CI = 1.14–1.87, *P* = 0.003) and CLP (OR = 1.58, 95% CI = 1.27–1.96, *P* = 5.50 × 10^−5^) in the additive model. However, there was no statistical association between rs4791331 and CPO (OR = 1.21, 95% CI = 0.93–1.59, *P* = 0.159), confirming different genetic background among them.

**Table 2:** Association study of rs4791331 and NSOC susceptibility

	Case			Control			Additive model	
	CC	CT	TT	CC	CT	TT	OR (95% CI)	*P*
NSOC	531	283	42	585	214	24	1.43 (1.20–1.70)	5.10 × 10^−5^
Subgroups								
CLO	160	91	11				1.46 (1.14–1.87)	0.003
CLP	224	129	21				1.58 (1.27–1.96)	5.50 × 10^−5^
CPO	147	63	10				1.21 (0.93–1.59)	0.159

*CLO* cleft lip only, *CLP* cleft lip and palate, *CPO* cleft palate only, *OR* odds ratio, *95% CI* 95% confidence interval, additive model: rs4791331 CC/CT/TT

### Effects of rs4791331 on cell apoptosis, cell proliferation, and the cell cycle

**Fig. 1:**
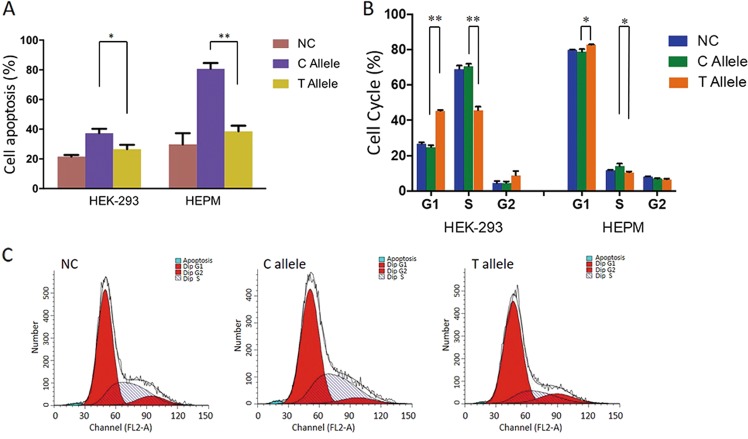
**a** Effects of rs4791331 C/T allele on cell apoptosis in HEK-293 and HEPM cells. The apoptosis of cells was detected by Annexin V-FITC/PI double staining method at 48 h after transfection with rs4791331-C, rs4791331-T, or nonsense control vector (NC). Results are shown as means ± SD from three experiments, each with three replicates. **b** Representative histograms depicting cell cycle profiles of cells transiently transfected with rs4791331-C/T allele or nonsense control vector, respectively. Cells were stained with PI and analyzed by flow cytometry. Various phases of the cell cycle in **c** HEK-293 and HEPM cells. The results are means of triplicate independent experiments. (*P *<* 0.05, **P < 0.01, ***P < 0.001.)

As shown in Fig. 1a, the expression of the T allele led to a significant decrease in cell apoptosis, indicating that rs4791331-T exerts an antiapoptotic effect (HEK-293: *P* = 0.011, HEPM: *P* = 0.007). However, neither the rs4791331 C nor T allele had an effect on the proliferation of HEK-293 and HEPM cells (Supplementary Fig. S4). Cell cycle distribution showed that cell counts in the G1 phase were significantly decreased in HEK-293 (*P* = 0.001) and HEPM (*P* = 0.011) cells transfected with the vector with the rs791331 T allele compared with those with the C allele, accompanied by a substantial accumulation of the cells in S phase (HEK-293: *P* = 0.001, HEPM: *P* = 0.013) (Fig. 1b, c). Therefore, the rs4791331 T allele may have an antiapoptotic effect due to the enhancement of cell cycle progression at the G1/S transition in cells.

### Nuclear protein binds preferentially to the T allele sequence at the rs4791331 locus

**Fig. 2:**
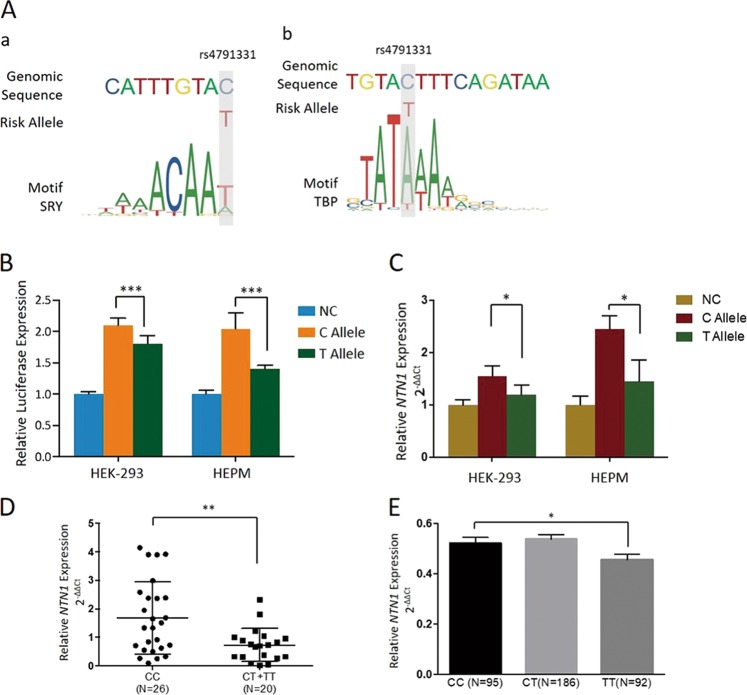
**a** The position of rs4791331 in Chr17p13.1 reside within TBP (a) and SRY (b) DNA-binding motifs. **b** The plasmid constructed with the fragments of *NTN1* containing the C or T allele of rs4791331 was transfected in HEK-293A cells and HEPM cells. A plasmid construct with a nonsense sequence was used as the negative control (NC). Binding ability assay of plasmids and transcription factors were detected. The firefly luciferase to Renilla luciferase ratio was considered the relative luciferase expression. **c** Quantitative real-time polymerase chain reaction analysis of *NTN1* expression at 48 h after plasmids transfection in HEK-293 and HEPM cells. Independent triplicate experiments were performed. Results are shown as mean values with the standard deviation (SD) normalized to *GAPDH*. **d** *NTN1* expression levels in 46 NSCL/P cleft lip tissue samples. qRT-PCR was used to test the *NTN1* mRNA expression in 46 cleft lip tissues (26 samples of CC genotype, 18 samples with CT genotype and 2 of TT genotype). The results were normalized to *GAPDH*. Error bars indicate the standard deviations. **e** The e-QTL result of rs4791331-*NTN1* in the lymphocytes of European population from 1000 Genome database. (*P *<* 0.05, **P < 0.01, ***P < 0.001)

JASPAR revealed that rs4791331 mapped within the binding motifs of homeodomain transcription factors with SRY and TBP. A binding preference to the T allele over the C allele was predicted (Fig. 2a).

### rs4791331 C > T affects the expression of *NTN1*

The luciferase reporter assay showed dramatic allelic differences in enhancer activity. The fragments with the T allele at rs4791331 showed significantly lower enhancer activity to drive luciferase gene expression in HEK-293 cells (*P* < 0.001) and HEPM cells (*P* < 0.001) than those with the C allele (Fig. 2b). Furthermore, compared with the C allele, the T allele was associated with lower expression of *NTN1* (HEK-293: *P* = 0.049, HEPM: *P* = 0.036, Fig. 2c).

### *NTN1* expression in human tissues and mouse craniofacial structures

A total of 46 cleft lip tissues from NSCL/P patients were obtained and genotyped to assess the mRNA expression of *NTN1* in vivo. The frequencies of the CC, CT, and TT genotypes of rs4791331 were 26, 18, and 2, respectively, in the samples. We found that the CT and TT genotypes were significantly associated with decreased expression of *NTN1* compared with the CC genotype (*P* = 0.014, Fig. 2d). In addition, *NTN1* expression in the lymphocytes of the European population from the 1000 Genome database showed that individuals with the TT genotype had lower *NTN1* expression than those with other genotypes (*P* = 0.047) (Fig. 2e).

Then, the relative expression of *Ntn1* in the 10.5–14.5-day mouse embryos (time period that lip and palate fusion occurred) was detected in the proximal maxillary process with an increasing trend from E10.5d to E13.5d and then a decrease on E14.5d. *Ntn1* expression in the distal maxillary process increased from E11.5d to E12.5d and then leveled off (Supplementary Fig. S5).

### Zebrafish phenotype

**Fig. 3:**
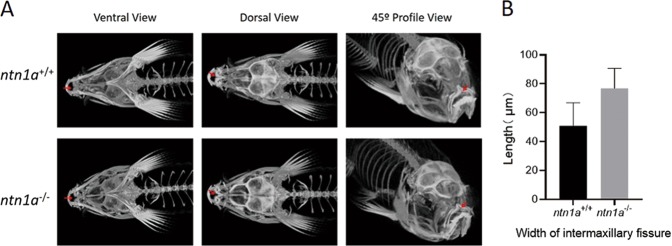
**a** The ventral, dorsal and 45° profile views of F2 generation zebrafish (4 months age) under Micro-CT (yellow array: mandibular width is the width between the inside border of the articulation; red array: intermaxillary fissure, the width were measured as the distance between the most anterior and inside point of the bilateral maxilla. *ntn1a*^−/−^: the homozygous knock out line, *ntn1a*^+/+^: the wild homozygous line). **b** The comparison of the width of mandibular and the width of intermaxillary fissure between *ntn1a*^+/+^ and *ntn1a*^−/−^ group. (**P < 0.01)

The *ntn1a* knockout F2 generation of zebrafishes all survived. Under micro-CT, the *ntn1a*^−/−^ mutant adult zebrafish at 4 months of age showed relatively wider intermaxillary fissures than the *ntn1a*^+/+^ zebrafish (Fig. 3, *P* = 0.057). Although neither cleft lip nor cleft palate was observed, the wider intermaxillary fissure indicates the putative function of *ntn1a* in orofacial fusion.

## Discussion

GWASs have identified several SNPs related to NSCL/P in recent years. Because the majority of these loci are situated within intergenic or intronic regions, a mechanistic understanding of how they contribute to phenotypes is limited. However, an increasing number of studies have demonstrated the important role of intronic mechanisms, such as altering RNA splicing activity [28], modulating RNA and protein expression [29] and changing the structure of proteins [30], which regulate the network for multifactorial diseases. A recent understanding of the control of gene expression has emerged as a key tool for connecting DNA sequence variation to phenotypes. A variant in the intronic region may alter the molecular mechanism of complex traits by affecting transcriptional activity through the altered binding ability of motifs. For instance, López Rodríguez et al. identified SNPs in a FOXA2-regulated transcriptional enhancer at a type 2 diabetes intronic locus that controls GCKR expression in liver cells [31]. For NSCL/P, Cvjetkovic et al. identified an SNP in intron 1 of *FZD6* that creates an allele-specific protein-binding site and decreases promoter activity, which is involved in craniofacial development and contributes to NSCL/P by perturbing the WNT signaling pathway [18].

Before the GWAS of NSCL/P, the *NTN1* gene in the chromosome 17p13 region was not associated with NSCL/P susceptibility. However, this region was shown to be related to NSCL/P risk by GWAS [11] and sequencing [17]. Notably, rs4791331 was once recognized as a “second hit” locus (*P* = 6.4100E−06) in Asian populations by Beaty et al. in a GWAS of case-parent trios of NSCL/P [7]. In this study, we hypothesized that rs4791331 in maximal LD with the lead SNP rs4791774 may be a functional SNP in this region according to the prediction of publicly available databases. In addition, the association study between rs4791331 and NSCL/P susceptibility confirmed that rs4791331 is associated

with the risk of NSCL/P, but no association between rs4791331 and CPO was found. Then, the mechanism was examined experimentally. According to the results of experiments in vitro, the mutant s4791331-T allele can downregulate the expression of *NTN1* probably by altering the motif binding ability in HEK-293 and HEPM cells, followed by the reduction of cell apoptosis and modulation of cell cycles. It indicated that variant of rs4791331 may be related to the risk of NSCL/P and indicated the distinct pathogenic mechanisms between CLO, CLP, and CPO [32].

*NTN1* is involved in various signaling pathways as a secreted protein that is expressed in the cell-extracellular matrix. For example, this protein is the effector molecule of IGF1/MEK/ERK and IGF1/ PI3K/AKT signaling pathways during cochlear hair cell protection [33] and modulates angiogenesis by the netrin-1/FAK/Src/CD151 signaling axis [34]. During embryonic development, *NTN1* was detected in multiple tissues, such as the nervous system, vasculature, lung, pancreas, muscle, and mammary gland [35], and functioned as the major cue for midline attraction of the nervous system [36], regulating the progression of semicircular canal morphogenesis in the inner ear [37]. Moreover, the reduction of *Ntn1* expression in mouse embryos led to a lack of white spots of milk in the stomach and death during the perinatal period, consistent with an orofacial cleft phenotype [17, 38]. However, its role in the pathogenic mechanism of NSCL/P has never been addressed. In our study, the *NTN1* gene variant was associated with modulation of cell apoptosis and the cell cycle. *NTN1* was shown to regulate cell apoptosis in colorectal tumorigenesis [39] and modulate the cell cycle in angiogenesis [34], but the specific molecular mechanism remains unclear.

In conclusion, we found that the C > T base change of rs4791331 resulted in abnormally low expression of *NTN1*, probably through disrupted motif binding, which may be involved in the development of NSCL/P. Our findings will help elucidate the etiology and genetic factors of NSCL/P.

## Supplementary information

Supplementary data
