# Supplementary material for: The functional variant of NTN1 contributes to the risk of nonsyndromic cleft lip with or without cleft palate
Source: Eur J Hum Genet. 2019 Nov 28;28(4):453–60. doi: 10.1038/s41431-019-0549-4 (PMC7080719; doi:10.1038/s41431-019-0549-4)
Supplement: Supplementary file 1 — Supplementary data [file 41431_2019_549_MOESM1_ESM.docx]

**The functional variant of *NTN1* contributes to the risk of Non-syndromic Cleft Lip with or without Cleft Palate**

Dandan Li^1^, Shu Lou^1^,Lan Ma^1^, Guirong Zhu^1^, Chi Zhang^1^, Yongchu Pan^1^ , Lin Wang^1^

^1^Jiangsu Key Laboratory of Oral Diseases, Nanjing Medical University, Nanjing, 210029, China

**Correspondence：**

Yongchu Pan

JiangSu Key Laboratory of Oral Diseases, Nanjing Medical University, 136 Hanzhong Road, Nanjing, 210029, China

E-mail: Panyongchu@njmu.edu.cn

Tel.: +86-25-86862025; Fax: +86-25-86862823

**Table S1.** The sequence of primers and probes.

| **Experiment** | **Description** | **Primer Sequence (5'-3')** | Probe |
| --- | --- | --- | --- |
| Genotyping | rs4791331 | F: CCATCAAGATAACATGAGTAACAATCC | C allele: FAM-TGTAAACATGAAAGTCTATTA-MGB |
|  |  | R: TGAGATGAGTTGTGCTTTTCATTAAGT | T allele: VIC-AAGTGTAAACATAAAAGTCTATTA-MGB |
| RT-qPCR | *NTN1* | F: ATGATGCGCGCAGTGTGG |  |
|  |  | R: TTCTTGCACTTGCCCTTCTT |  |
| RT-qPCR | *GAPDH* | F: GCACCGTCAAGGCTGAGAAC |  |
|  |  | R: TGGTGAAGACGCCAGTGGA |  |

RT-PCR: quantitative reverse transcriptase-polymerase chain reaction

**Table S2.** Exon1 of *ntn1a* gene（The red letters mark the recognition sequence, the letter highlighted yellow were the target sequence）:

ATGTTGAGAGTCTCTGATGCTTTGGTCACTTTGGTGACTCTCTGCTGTGTGCTCAAAGGGACTGTCGGCGGATATGGAATGAGCATGTTCGCCGCTCAGACCTCCCCGCCGGATCCGTGTTACGACGAGAACGGACACCCCAGAAGATGCATCCCCGACTTCGTAAACGCGGCGTTCGGGAAAGAAGTACGCGCGTCCAGCACCTGCGGCAAAACGCCGAGTCGTTACTGCGTGGTGACCGAGAAAGGGGACGAAAGACACAGAAACTGCCACACGTGCGACGCGTCAGACCCAAAGAAGAATCACCCACCAGCTTACCTGACCGACCTGAACAATCCTCACAATCTCACCTGCTGGCAGTCGGACAATTACCTCCAGTATCCTCAAAACGTCACTTTAACTTTATCCTTGGGCAAGAAATTTGAGGTGACCTACGTGAGTTTGCAGTTCTGCTCACCTCGACCGGAGTCTATGGCGATCTTTAAATCGATGGACTACGGAAAGTCCTGGGTGCCTTTCCAGTACTACTCGACCCAGTGTAGAAAGATGTACAACAAGCCCAGCAAAGCCACGATTACTAAGCAGAACGAGCAAGAGGCCATCTGCACAGATTCTCACACCGACATGCATCCTCTCTCCGGCGGGCTGATCGCGTTCAGCACCCTGGACGGGCGACCCTCCGCGCACGACTTTGACAATTCACCCGTACTTCAGGACTGGGTGACCGCCACTGACATTAAGGTGACTTTCAGCCGCCTGCACACTTTCGGAGACGAAAACGAGGATGACTCGGAGCTGGCCAGAGATTCCTATTTTTACGCAGTTTCCGACCTGCAGGTTGGAGGCAGATGTAAGTGTAATGGACACGCATCACGGTGCGTCAAAGACCGGGATGGAAACCTAGTGTGCGAGTGCAAGCACAACACAGCCGGACCAGAGTGTGACAGATGCAAACCTTTTCACTATGACCGACCCTGGCAGCGCGCAACCGCCAGAGAAGCCAACGAATGTGTCG

**Table S3.** The prediction of the SNP function by silico analysis

| rs No. | r^2^ with rs4791774 | Regulome DB score | F-SNP functional score ^a^ |
| --- | --- | --- | --- |
| rs4791774 | - | 4: minimal binding evidence | 0.194 |
| rs4791331 | 0.99 | 2b: likely to affect binding | 0.714 |
| rs9891446 | 0.8 | no result | 0.5 |
| rs36047638 | 0.8 | 6: minimal binding evidence | 0.28 |

^a^ The score range from 0 to 1. The higher score indicate the great functional significance.


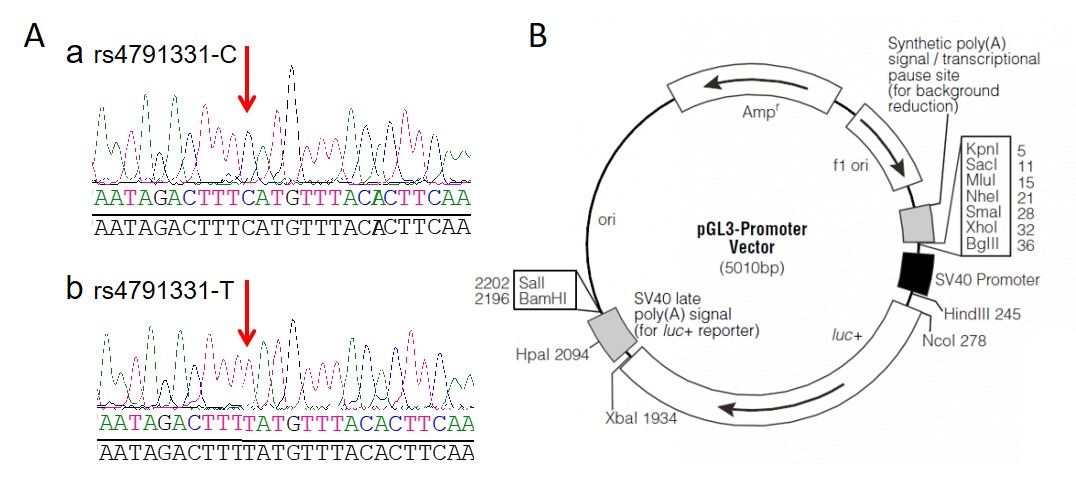


**Figure S1.** Result of sequencing showed that fragment containing rs4791331 C allele or T allele (A) was successfully synthesized and inserted into PGL3-promotor vector（B）.


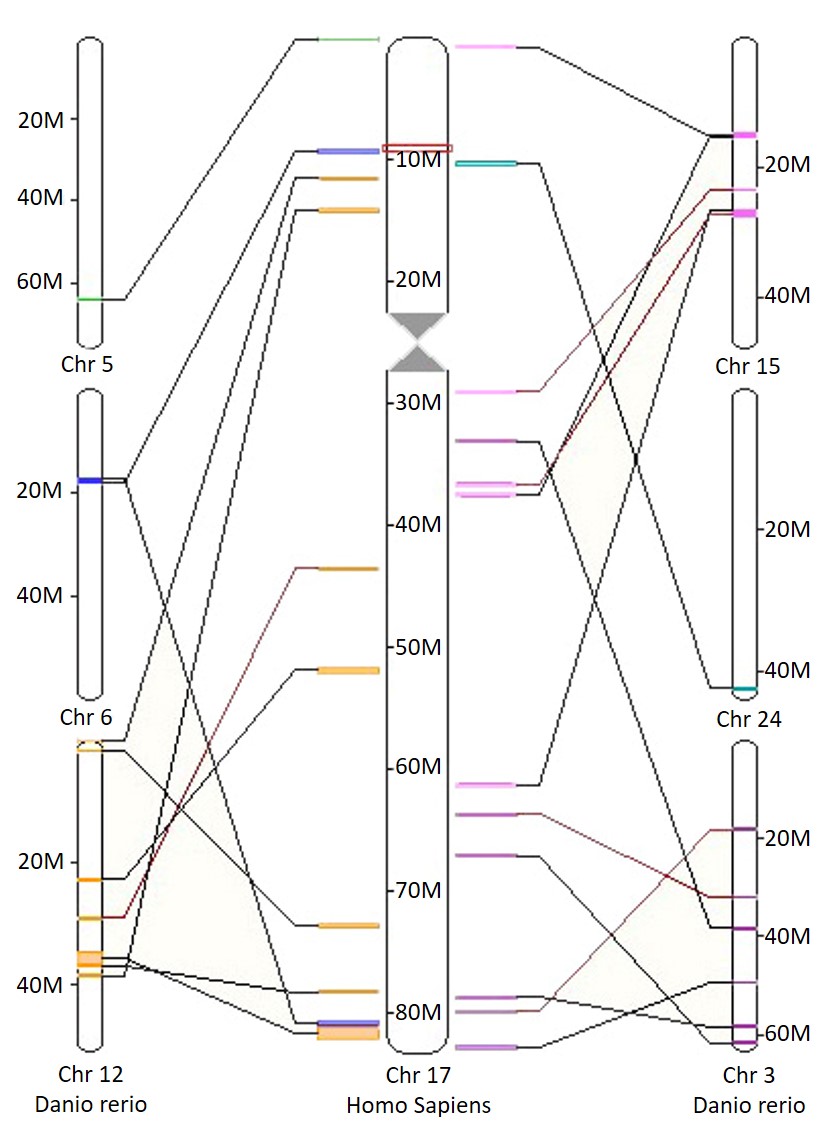


**Figure S2.** The Synteny analysis between human chromosome 17 and Zebrafish. The red frame in human Chr17 shows the region of p13.1 containing *NTN1* gene.


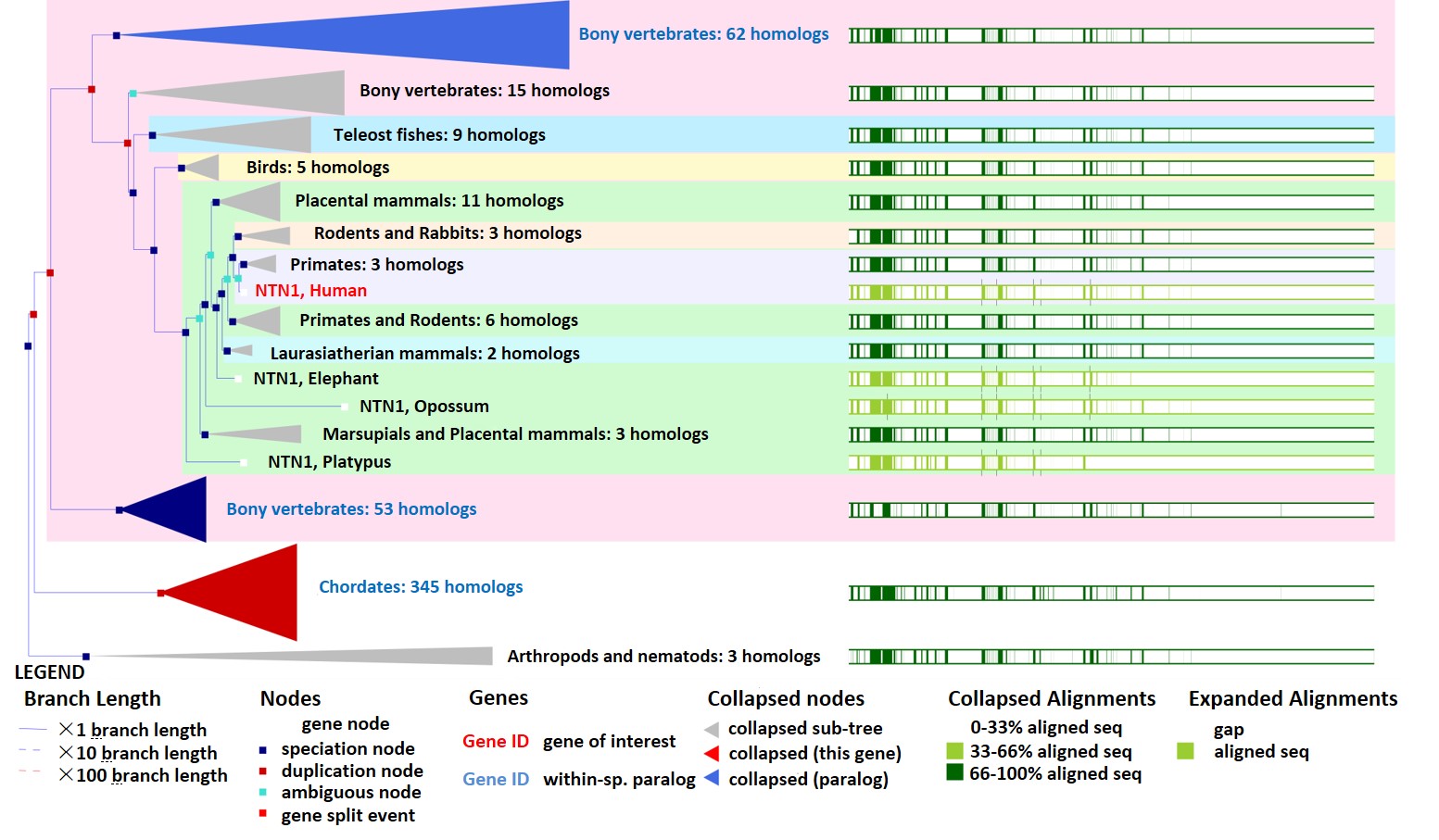


**Figure S3.** The phylogenetic tree analysis of human *NTN1* gene.

**Figure S4.** Effects of rs4791331 C/T allele on cell proliferation in HEK-293 and HEPM cells.


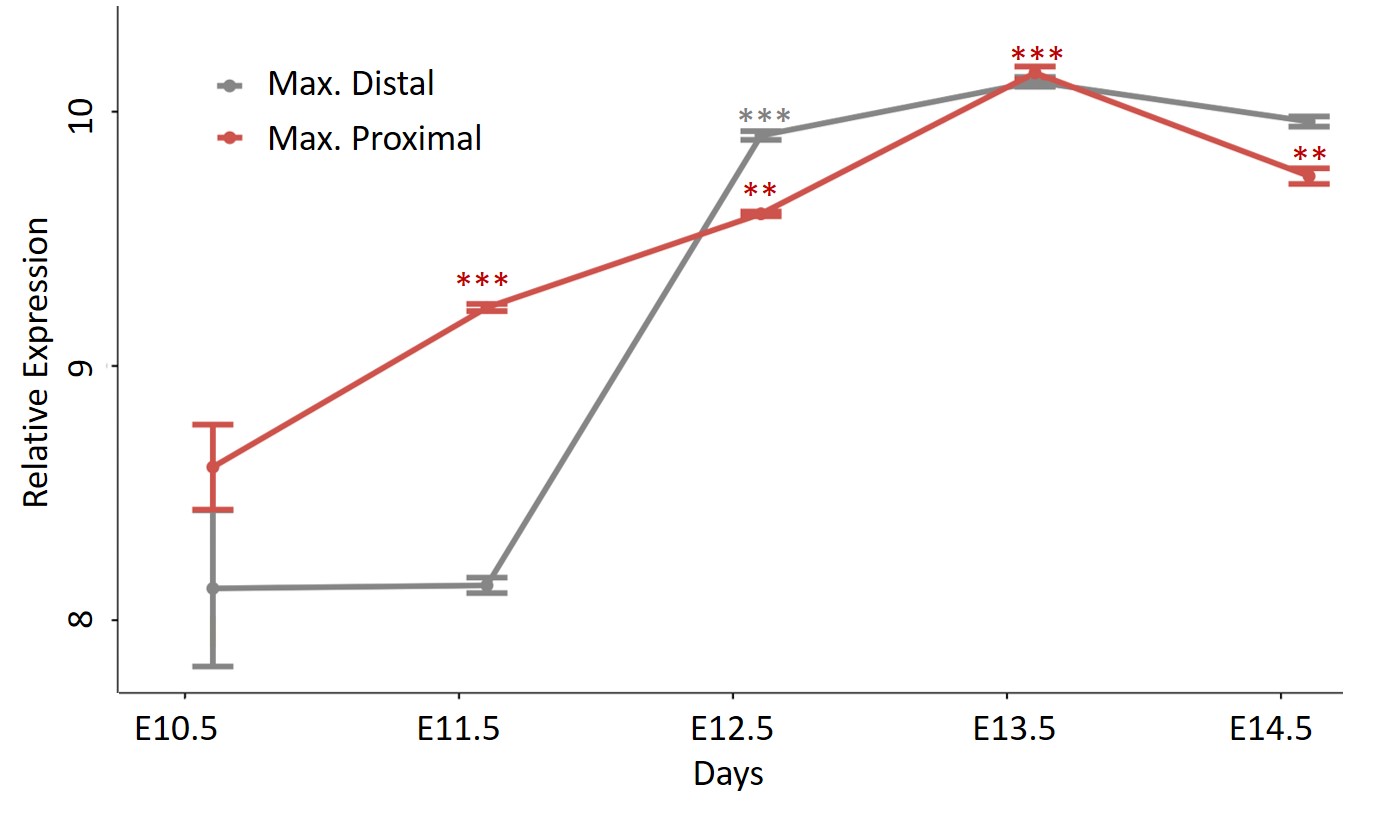


**Figure S5.** The relative expression of Ntn1 in the embryo 10.5-14.5 day of mouse. The raw microarray data was available at Gene Expression Omnibus (GEO, http://www.ncbi.nlm.nih.gov/geo/, accession number GSE55965 and GSE67985). (**P* < 0.05, ***P* < 0.01, ****P* < 0.001 in the multiple comparison of one-way ANOVA analysis compared with the *Ntn1* expression of the day before)
